# A special issue of *Essays in Biochemistry* on current educational developments in molecular bioscience

**DOI:** 10.1042/EBC20220032

**Published:** 2022-04-29

**Authors:** Luciane V. Mello, Helen R. Watson

**Affiliations:** 1 School of Life Sciences, University of Liverpool, U.K.; 2 Peninsula Medical and Dental Schools, University of Plymouth, U.K.

**Keywords:** assessment and feedback, bioscience education, innovative teaching, online teaching, skills development

## Abstract

The 4th joint UK Biochemical Society and Federation of European Biochemical Societies (FEBS) education event, *‘Evolving Molecular Bioscience Education’* took place online on May 27 and 28, 2021. The event, continuing the biennial series, comprised the invited speakers’ talks, group discussions and other participants’ pre-recorded flash presentations. Although the UK dominated, there were also speakers and participants from other European countries and other continents. This special issue includes a varied collection of articles written by the speakers and other participants.

This issue of *Essays in Biochemistry* comprises a varied collection of articles written by bioscience educators working in higher education across the world. The idea for the issue emerged from the 4th in the series of education training events run as a collaboration between the UK Biochemical Society and FEBS (Federation of European Biochemical Societies). This event, ‘Evolving Molecular Bioscience Education’ held in May 2021 saw a network of educators gather online to discuss advances and challenges in bioscience education, sharing good practice and making new connections. Delegates and speakers discussed a range of important topics in higher education, including how best to deliver transferable skills training, assessment and feedback, and innovative teaching approaches including developments in online learning. Many interesting and exciting ideas were shared by colleagues from around the world, leaving us with plenty of things to try out in our own real and virtual classrooms, and eager to collaborate with new and existing contacts in educational research. We thought we would like to capture some of these ideas to share with the wider molecular bioscience education community, and this is what we have done in this special issue of *Essays*.

Of course the COVID-19 pandemic has had an enormous and potentially long-lasting impact on education in general. In terms of molecular bioscience higher education, perhaps the most immediately obvious challenge was how to train our students in practical laboratory skills, when they could not leave their student accommodation. Virtual labs are something that colleagues in our field have been developing for a long time now (far pre-dating COVID-19) and became even more prominent during the pandemic, to allow continuation of practical skills development. In addition, the development of other online teaching approaches has been catalysed by the need for distance learning. Online courses to teach computational skills and refinement of blended classroom approaches have been successful in engaging students working from home. The high proportion of papers on online and digital learning in this issue perhaps reflects the increased focus this theme has received in light of the pandemic. Papers by Smith and Francis [1], Pal et al. [2], Unsworth and Posner [3], Cassambai et al. [4] and Sozmen [5] in this issue reflect some of the interesting developments in this area. We hope that as normality gradually returns, some successful aspects of online and digital education will be retained and used to engage and enthuse students about our subject.

Our molecular bioscience graduates are entering an exciting, varied and demanding labor market. Long gone are the days where academics need only to train students to follow in their own footsteps to one day run their own research groups. We need to help our students discover the wide range of futures they can lead, enable them to develop the vital transferable skills they will need and, potentially most importantly, help them understand what skills they have and how they can apply and grow these over time. Enabling students to develop their skills is a vital part of higher education, and it is important that as we create and develop programmes, we put skills at the heart of our curricula. The paper by Lund and Clyne [6] in this issue discusses aspects of skills development.

Innovation in molecular biosciences education is demonstrated and evaluated in many papers in this issue, but some focus specifically on teaching key concepts in a completely different way. As we have seen, particularly over the past two years, lectures and practicals aren't the only way to teach our subject. We hope that the papers by Austin et al. [7] and Sahai and Ivanova [8] in this issue inspire colleagues to try new ways of teaching the same subjects, to enthuse both their students and themselves! Their work also illustrates ways of fostering students' creativity and skills development.

Setting fair and rigorous assessments and delivering constructive feedback are both challenges that most in higher education face regularly. Large student cohorts and online delivery are just two factors that have required us to be more creative with our assessment strategies in recent years. Ensuring that the feedback we give to students can be used to help them improve is key, and providing formative feedback is important to aid student development and feedback literacy. Papers by Menon and Clyne [9] and Ray et al. [10] in this issue discuss student perspectives on various forms of feedback.

The papers in this issue comprise Perspectives and Case Studies and are designed to appeal to a range of audiences, including educators, students and interested colleagues from outside higher education. The Perspective articles aim to inform the reader about a relatively broad topic in molecular bioscience education and we hope they will be especially useful to those coming into a new field in education, or indeed new educators, as well as well-established educators looking to learn more. The case studies are reports written by colleagues to present preliminary evaluation of an educational approach or technique that they have introduced in their own classrooms. The case studies share the advantages and potential pit-falls of various educational approaches and we hope they will be useful for readers who are looking to implement something in their own teaching, and would like to find out more about the practicalities and pick up some tips.

We hope that you enjoy this issue of *Essays in Biochemistry* and you are very welcome to join us at our next Bioscience education event.

## Open Access

Open access for this article was enabled by the participation of University of Liverpool in an all-inclusive *Read & Publish* agreement with Portland Press and the Biochemical Society under a transformative agreement with JISC.

## Abbreviation

FEBS: Federation of European Biochemical Societies
